# State-Based Fault Diagnosis of Finite-State Vector Discrete-Event Systems via Integer Linear Programming

**DOI:** 10.3390/s25051452

**Published:** 2025-02-27

**Authors:** Qinrui Chen, Mubariz Garayev, Ding Liu

**Affiliations:** 1 School of Electro-Mechanical Engineering, Xidian University, Xi’an 710071, China; xidianchenqinrui@163.com; 2 Department of Mathematics, College of Science, King Saud University, Riyadh P.O. Box 2455, Saudi Arabia; mgarayev@ksu.edu.sa

**Keywords:** vector discrete-event system, fault diagnosis, K-diagnosability, predicate, integer linear programming

## Abstract

This paper presents a state-based method to address the verification of K-diagnosability and fault diagnosis of a finite-state vector discrete-event system (Vector DES) with partially observable state outputs due to limited sensors. Vector DES models consist of an arithmetic additive structure in both the state space and state transition function. This work offers a necessary and sufficient condition for verifying the K-diagnosability of a finite-state Vector DES based on state sensor outputs, employing integer linear programming and the mathematical representation of a Vector DES. Predicates are employed to diagnose faults in a Vector DES online. Specifically, we use three different kinds of predicates to divide system state outputs into different subsets, and the fault occurrence in a system is detected by checking a subset of outputs. Online diagnosis is achieved via solving integer linear programming problems. The conclusions obtained in this work are explained by means of several examples.

## 1. Introduction

As discrete-event systems (DESs) grow increasingly complex, fault diagnosis becomes a challenging endeavor that cannot solely resort to trial-and-error or experience. Adopting a systematic approach is vital for efficiently addressing diagnostic challenges. Diagnosability in DESs, as studied in prior works [1,2], refers to the probability to detect a fault within a limited delay once it occurs. Fault detection acts as a monitoring mechanism, revealing potential faults by observing the behavior of a DES.

The formal definition of diagnosability for DESs is initially introduced in [3], where a fault detection method based on events is introduced for regular languages that are represented by finite-state machine (FSM) models [4]. Wonham and Lin [5,6] touch upon the supervisory control problem of partially observed FSMs. A diagnoser is constructed to detect a set of possibly occurring faults [3] or a collection of fault states that a system can reach [7] after an observed event. A diagnoser is used to extend the diagnosability concept to stochastic automata in [8] and to the decentralized case in [9,10]. A coordinated decentralized architecture consisting of local sites communicating with a coordinator that is responsible for diagnosing the failures occurring in a DES is proposed in [9]. The study in [10] presents a model-based technique for the diagnosis of large-scale active systems that are treated as distributed DESs with events being received asynchronously. In the literature, the diagnosability concept is extended to the centralized cases [11,12,13,14], to the decentralized cases [9,10,15], and to the distributed cases [16,17].

The problem of the verification of diagnosability has also been solved within the framework of Petri nets (PNs) [18,19] with two representations: graphical and mathematical. In [20], assuming that not every place of a PN is observable, every transition is unobservable, and a fault is represented by a transition, a diagnoser is constructed to verify the diagnosability of the system. One may determine whether a model is a member of a specified net subclass by the graphical representation of a PN. High-efficiency algorithms are developed for verifying the diagnosability of a specified PN subclass by investigating its peculiarity. Assuming that all places are not observable, the diagnosability of a PN is verified by constructing its diagnoser in [21]. The mathematical representation of a PN makes it possible to verify its diagnosability using common mathematical techniques like integer linear programming (ILP). The *K*-diagnosability of a PN is verified via ILP in [22]. An approach [23] using ILP achieves online diagnosis by recording observed transition sequences.

In general, only partial observation of events and states can be obtained since real systems have a limited number of sensors. Modeling faults as unobservable state transitions [20,24,25] or unobservable events [3,26] is one of the fault models that has been studied extensively. Fault diagnosers of DESs or PNs are constructed to verify the fault diagnosability or diagnose faults in some research based on states (i.e., state-based) [27,28], or markings in observed places [29,30]. In [27], a state-based methodology for fault diagnosis in a fully observable DES is introduced. This approach assumes a categorization of the system state set, distinguishing between failure and normal states. The objective of the diagnosis problem is to ascertain whether the current state is faulty or normal upon receiving the most recent observation.

Compared with traditional automata and PNs, a vector discrete-event system (Vector DES) offers greater flexibility. This structured model is particularly effective for representing systems with inherent additive structures, such as smart manufacturing systems. Vector DESs provide a modeling approach, particularly to systems that contain groups of entities with the same characteristics. When the internal system shares similar structural characteristics, it is logical to improve the model of an abstract automaton by leveraging its regularity in algebra. Compared to Petri nets (PNs), Vector DESs offer the advantage of more compact modeling. Within the framework of Vector DESs, PNs are simply used as a graphical tool for illustration, and the mathematical theory of PNs is not utilized [31,32,33,34,35]. This is because the primary aim of developing Vector DESs is to apply Ramadge–Wonham (R-W) methods to structured systems with vector addition. Moreover, since a state vector of a Vector DES is composed of integers, the definition of states becomes more flexible, and it is also crucial for the state feedback control [35]. An event-based approach to verifying the diagnosability property of Vector DES is presented in [31]. Particularly, predicates are proposed to partition system states into different subsets, based on which the fault diagnosability is verified.

This research investigates the problem of state-based K-diagnosability analysis and state-based fault diagnosis in a finite-state Vector DES, particularly in scenarios where sensors are embedded within specific state components. The idea of this paper is generated to characterize all the state output sequences corresponding to an event sequence $u\varepsilon_{f}v$, where *v* sustains the system evolution after the fault $\varepsilon_{f}$ occurs, while *u* enables a fault $\varepsilon_{f}$ from the initial state. The following is a summary of this work’s primary contributions.

1. We first investigate and formulate the definition of the state-based K-diagnosability in a Vector DES. For the verification of state-based K-diagnosability, a necessary and sufficient condition is introduced.
2. A standard mathematical tool is utilized to verify state-based K-diagnosability and diagnose, preventing a full state enumeration. The presented method does not depend on the construction of a diagnoser, because it solves ILP problems to verify the state-based K-diagnosability and achieve online diagnosis.

The rest of this paper is arranged as follows. Section 2 provides the preliminaries on system models. Section 3 describes the problem statement, together with the definitions of the state-based diagnosability and state-based K-diagnosability. For the verification of state-based K-diagnosability, a necessary and sufficient condition is introduced in Section 4. Section 5 develops an algorithm to achieve online diagnosis. An example of a production line repairing damaged parts is presented to illustrate the proposed methods in Section 6. In the end, conclusions are provided in Section 7.

## 2. Preliminaries

A DES plant is a *generator* $G=(D,\Sigma ,\xi ,d_{0},D_{m})$, where *D* stands for the state set, Σ for the alphabet, ξ: D×Σ→D for the (partial) transition function, $d_{0}$ for the initial state, and $D_{m}\subseteq D$ for the marker state set. $\Sigma^{+}$ and $\Sigma^{*}$ are used to represent the set of all finite sequences of symbols defined over Σ and the Kleene closure of Σ, respectively, i.e., $\Sigma^{*}:=\Sigma^{+}\cup \left\{\epsilon \right\}$, where $\Sigma^{+}=\Sigma \cup \Sigma^{2}\cup \Sigma^{3}\cup \cdots$, and ϵ is the empty sequence [35]. An element $w\in \Sigma^{*}$ is called a *string*, with |w| representing the length of *w*. Given σ∈Σ, we follow the notations in [35], and use ξ(d,σ)! to represent that the transition ξ(d,σ) is defined. The *closed behavior* of *G* is

(1) $$L\left(G\right):=\{w\in \Sigma^{*}|\xi \left(d_{0},w\right)!\},$$

and the *marked behavior* is

(2) $$L_{m}\left(G\right):=\left\{w\in L\left(G\right)|\xi \left(d_{0},w\right)\in D_{m}\right\}\subseteq L\left(G\right).$$

Given a DES *G*, a state d∈D is *reachable* if there exists a string $w\in \Sigma^{*}$ such that $\xi (d_{0},w)!$ and $d=\xi (d_{0},w)$. For any state d∈D,

(3) $$L\left(G,d\right):=\{w\in \Sigma^{*}|\xi \left(d,w\right)!\}.$$

Define

(4) $$L/w:=\{v\in \Sigma^{*}|w\in L\left(G\right),wv\in L\left(G\right)\}.$$

The space of *n*-vectors (i.e., ordered *n*-tuples) with components in Z (resp., N) is represented by $Z^{n}$ (resp., $N^{n}$), where Z and N stand for the sets of integers and natural numbers, respectively. The “direct sum” operation ⨁ is used to form structures similar to $N^{n}⨁Z^{m}$ or $Z^{n}⨁Z^{m}$. A Vector DES [33,34,35] is defined as $G=(D,\Sigma ,\xi ,d_{0},D_{m})$, but with a vector structure. Generally speaking, $D!=N^{n}⨁Z^{m}$, while ξ: D×Σ→D satisfies the following form:

(5) $$\xi \left(d,\sigma \right)=d+e_{\sigma },$$

where $e_{\sigma }\in Z^{n+m}$ and σ∈Σ. Let $\Sigma =\{\sigma_{1},\ldots ,\sigma_{k}\}$ and write $e_{i}$ for $e_{\sigma_{i}}$. Define

(6) $$E:=\left[e_{1}\ldots e_{k}\right]\in Z^{p\times k}$$

as the displacement matrix for **G**. Let |D| represent the size of state set.

Let $E(\cdot ,e_{i})$ represent the column of *E* corresponding to event $e_{i}$. Define

(7) $$V:\Sigma^{*}\to N^{k}:w\mapsto \left[v_{1}\left(w\right)\ldots v_{k}\left(w\right)\right]\in N^{k\times 1},$$

where $v_{j}\left(w\right)$ is the number of occurrences of $\sigma_{j}$ in *w*. A more general definition of a Vector DES would add the following enabling conditions. Assume $D=N^{n}$. Given $e_{\sigma }\in Z^{n}$ and $f_{\sigma }\in N^{n}$, let $e_{\sigma }:=e_{\sigma }^{+}-e_{\sigma }^{-}$, $f_{\sigma }:=e_{\sigma }^{-}$, where $e_{\sigma }^{+},e_{\sigma }^{-}\in N^{n}$. Define

(8) $$E^{+}:=\left[e_{1}^{+}\ldots e_{k}^{+}\right]\in N^{p\times k}$$

and

(9) $$E^{-}:=\left[e_{1}^{-}\ldots e_{k}^{-}\right]\in N^{p\times k}.$$

**Figure 1:**
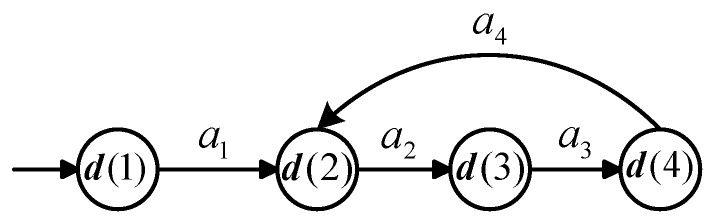
Vector DES model in Example 1.

**Example** **1.** *Consider a Vector DES $G=(D,\Sigma ,\xi ,d_{0},D_{m})$ in Figure 1, where the state vector is $d:={\left[d\left(1\right),d\left(2\right),d\left(3\right),d\left(4\right)\right]}^{T}\in N^{4\times 1}$, alphabet is $\Sigma =\{a_{1},a_{2},a_{3},a_{4}\}$, and the initial state is $d_{0}={\left[1,0,0,0\right]}^{T}$.*

*Displacement matrix $E\in Z^{4\times 4}$ and $E^{-}\in Z^{4\times 4}$ are given as follows:*

$$\begin{array}{cc} & \begin{array}{cccc} a_{1} & a_{2} & a_{3} & a_{4} \end{array}\\ \begin{array}{c} d(1)\\ d(2)\\ d(3)\\ d(4) \end{array} & \left(\begin{array}{cccc} -1 & 0 & 0 & 0\\ 1 & -1 & 0 & 1\\ 0 & 1 & -1 & 0\\ 0 & 0 & 1 & -1 \end{array}\right) \end{array}$$

$$\begin{array}{cc} & \begin{array}{cccc} a_{1} & a_{2} & a_{3} & a_{4} \end{array}\\ \begin{array}{c} d(1)\\ d(2)\\ d(3)\\ d(4) \end{array} & \left(\begin{array}{cccc} 1 & 0 & 0 & 0\\ 1 & 1 & 0 & 1\\ 0 & 1 & 1 & 0\\ 0 & 0 & 1 & 1 \end{array}\right) \end{array}$$

*All states of* ***G*** *are listed in Table 1.*

Consider a Vector DES $G=(D,\Sigma ,\xi ,d_{0},D_{m})$. A predicate *P*, as defined in [33,34], operates on D and is represented as a function P:D→{0,1}. In essence, *P* can be viewed as denoting the corresponding state subset associated with it, i.e.,

(10) $$D_{P}=\left\{d\in D|P\left(d\right)=1\right\}\subseteq D.$$

P(d)=1 is often written as d⊧P (“d satisfies *P*”). For the Vector DES **G** presented in Example 1, a predicate *P* can be defined as

$$D_{P}=\left\{d\in D|d\left(1\right)=0\right\}$$

or equivalently,

d⊧Piffd(1)=0.

## 3. Problem Formulation

States and events may be partially observable in a real system that can be modeled by a Vector DES. This paper considers the scenario in which all events of a Vector DES are unobservable and the states are partially observable. Alternatively, this setup implies that sensors are solely equipped in specific components of a state, which has been studied in the framework of state-based partially observed automata [27,36]. Consider a Vector DES designated for diagnosis, modeled as a reachable deterministic finite-state Moore automaton $G=(D,\Sigma ,\xi ,d_{0},Y,\lambda )$. Here, D, Σ, and Y represent the finite state, event, and output sets, respectively. The notation $d_{0}$ denotes the initial state, ξ stands for the transition function, and λ: D→Y is defined as the output map [27] (this output map can be formulated by taking into account the requirements of a specific physical system). Formally, suppose that a state d∈D’s component elements are separated into two sets: observable components set $d_{o}$ and unobservable components set $d_{uo}$, and $D=D_{o}⨁D_{uo}$.

We shall concentrate on the diagnosability and diagnosis of a single fault $\varepsilon_{f}$ instead of the diagnosability and diagnosis of a type of fault without sacrificing generality. Set $D=D_{n}\cup D_{f}$ can be classified into a normal state subset $D_{n}$ and faulty state subset $D_{f}$ including the states that are reachable from $d_{0}$ via a fault event represented by $\varepsilon_{f}$.

**Definition** **1** (Output projection)**.** *Given a Vector DES $G=(D,\Sigma ,\xi ,d_{0},D_{m})$, $D=D_{o}⨁D_{uo}$, and $d={\left[d\left(1\right),d\left(2\right),\ldots ,d\left(k\right)\right]}^{T}\in D\;\left(k\in N\right)$, the output projection $Pr:D\to D_{o}$ is defined as*

(11) $$Pr\left(d\right)={\left[d\left(i\right)\right]}^{T},d\left(i\right)\in d_{o}\;\left(i\leq k\in N\right).$$

The output projection Pr just “erases” the unobservable state components of a state.

**Example** **2.** *Continuing to Example 1, we briefly explain the output projection as follows. Assume $d_{uo}:=\left\{d\left(4\right)\right\}$. For instance, given $d_{1}={\left[0,1,0,0\right]}^{T}$, it comes $Pr\left(d_{1}\right)={\left[0,1,0\right]}^{T}$.*

**Definition** **2.** *Given a Vector DES G, a vector $h\in N^{n}$ is called an E-invariant if E·h=0.*

Similar to the definition of the *support* of a T-invariant in net theory [22], we define the *support* of an E-invariant h as the set of events that corresponds to entries of h that are not zero. If no suitable nonempty subset of a support can be a support, then the support is considered as being minimal. T(G) stands for the set of minimal support E-invariants of G.

In this work, the output map is defined as the output projection and Pr: D→Y. From now on, let us write a reachable nondeterministic finite-state Moore automaton $G=(D,\Sigma ,\xi ,d_{0},Y,Pr)$ in place of $G=(D,\Sigma ,\xi ,d_{0},Y,\lambda )$. We adopt the following assumptions in the remainder of the paper.

**Assumption** **1.** *The state set of a Vector DES can be classified according to the condition (normal or failure) of the system.*

**Assumption** **2.** *The system G is deadlock-free, i.e., (∀d∈D)(∃σ∈Σ)ξ(d,σ)!*

**Assumption** **3.** *The fault modes are permanent, i.e., the system stays in the faulty condition indefinitely after a fault occurs.*

**Assumption** **4.** *The initial state $d_{0}$ is unique.*

The notion of diagnosability guarantees that, upon the occurrence of a fault in a system, it must be detected within a limited delay. The formal descriptions of *state-based diagnosability* and *state-based* K-*diagnosability* are presented in what follows.

**Definition** **3.** *Given a Vector DES $G=(D,\Sigma ,\xi ,d_{0},Y,Pr)$ and a fault $\varepsilon_{f}$, G is state-based diagnosable if*

(12) $$\begin{array}{c} \left(\exists h\in N\right)\left(\forall d_{f}\in D_{f}\right)\left(\forall v\geq h\right)\left(\forall d_{e}^{v}\in X\left(d_{f},v\right)\right)d_{e}^{v}\subseteq D_{f}, \end{array}$$

*where $X(d_{f},v)$ represents the set of all state estimations provided after v events occurring from $d_{f}$.*

Let $d_{f}$ be any faulty state. Condition (12) indicates that there exists h∈N such that, once v≥h, the set of all state estimations provided after *v* events occurring from $d_{f}$ is the subset of the faulty state subset $D_{f}$.

**Definition** **4.** *Given a Vector DES $G=(D,\Sigma ,\xi ,d_{0},Y,Pr)$, a fault $\varepsilon_{f}$, and K∈N, G is state-based K-diagnosable if*

(13) $$\begin{array}{c} \left(\forall d_{f}\in D_{f}\right)\left(\forall v\geq K\right)\left(\forall d_{e}^{v}\in X\left(d_{f},v\right)\right)d_{e}^{v}\subseteq D_{f}, \end{array}$$

*where $X(d_{f},v)$ represents the set of all state estimations provided after v events occurring from $d_{f}$.*

The notion of K-diagnosability provides a means to specify an upper limit on the number of events required to detect a fault. If a system is considered to be state-based K-diagnosable, it implies that the system is also state-based diagnosable. However, the converse is not necessarily true. According to Definitions 3 and 4, if a system is state-based diagnosable, then there exists another integer value $K^{\prime }$ for which the system is also state-based $K^{\prime }$-diagnosable.

The fault detection problem discussed in this work employs a sequence of state output $(y_{1}y_{2}y_{3}\cdots$) to determine the condition of a Vector DES. It should be noted that it is assumed that just alterations in the output are observable. It indicates that, if the system goes from one state to another state with an identical state output, such transition will be unnoticed, i.e., the transition is not observable. In an output sequence, $y_{i}\neq y_{i+1}$ for i≥1. Given an output y, an upper limit for the number of states corresponding to the output y is given by H, which can be computed by Netlab [37]. For instance, in Example 2, we can obtain that H=2, implying that one state output corresponds to up to two system states.

Given a finite-state Vector DES $G=(D,\Sigma ,\xi ,d_{0},Y,Pr)$ and a fault $\varepsilon_{f}$, our goal is to realize the online diagnosis of a Vector DES by checking the subsets of states. When a new state output is generated, whether the faults occurred can be derived, the system can be in only one of the following states: normal, faulty, and uncertain. Enhancing the fault detection algorithm’s efficiency can be achieved at the expense of a slight rise in memory usage. Therefore, we just record the current state output. Of course, if we record the complete state output sequence, there will be a more accurate diagnosis decision. We need to find a specific predicate for a Vector DES such that, if a current state output satisfies this specific predicate, we can detect the fault. The primary issues to be investigated in this work are presented as follows.

**Problem** **1.** *Given a finite-state Vector DES G=(D,Σ,ξ, $d_{0}$, Y,Pr), $D\subseteq N^{n}$, and a fault $\varepsilon_{f}$, verify the state-based K-diagnosability of* ***G*** *via solving an ILP problem.*

**Problem** **2.** *Given a finite-state Vector DES G=(D,Σ,ξ, $d_{0},Y,Pr)$, $D\subseteq N^{n}$, and a fault $\varepsilon_{f}$, find a predicate P for* ***G*** *such that, if the current state output of the system satisfies P, one can determine the occurrence of $\varepsilon_{f}$.*

## 4. Verification of State-Based K-Diagnosability

### 4.1. Necessary and Sufficient Condition for State-Based K-Diagnosability of Finite-State Vector DESs

The primary outcome discussed in this section is to provide a necessary and sufficient condition for state-based K-diagnosability of a fault in a Vector DES. This condition is established as the solution to an ILP problem. Prior to introducing Lemma 1, a brief review of fundamental Petri net concepts and additional notations is provided.

A *Petri net* (PN) is a four-tuple $N=(\hat{P},T,Pre,Post)$, where $\hat{P}$ is a finite set of *places*, *T* is a finite set of *transitions*, *Pre*$:\hat{P}\times T\to N$, and *Post*$:\hat{P}\times T\to N$. A *marking* of a PN is a vector m: $\hat{P}\to N$. For further information on the fundamental concepts of PNs, the reader is directed to [22].

**Lemma** **1** ([38])**.** *Given a PN $\left(N,m_{0}\right)$ with a marking m and a sequence $\sigma \in T^{*}$, there exists a set of ρ non-negative integer vectors $w_{1},\ldots ,w_{\rho }$ with ρ≤|σ| such that the linear constraints listed below are satisfied*

(14) $$\left\{\begin{array}{c} m\geq \mathrm{Pre}\cdot w_{1},\\ m+C\cdot w_{1}\geq \mathrm{Pre}\cdot w_{2},\\ \cdots ,\\ m+C\cdot \sum_{i=1}^{\rho -1}w_{i}\geq \mathrm{Pre}\cdot w_{\rho },\\ \sum_{i=1}^{\rho }w_{i}=\sigma , \end{array}\right.$$

*if and only if σ is enabled at the marking m.*

A finite-length transition sequence that is enabled at a marking m in a PN must meet the necessary and sufficient condition given by Lemma 1. Based on Lemma 1, Lemma 2 is presented.

**Lemma** **2.** *Given a finite-state Vector DES $G=(D,\Sigma ,\xi ,d_{0},Y,Pr)$ and $D\subseteq N^{n}$, an output sequence $y_{1}y_{2}\cdots y_{\gamma }$ is generated at the initial state $d_{0}$, if and only if there exist $H^{2}\cdot \gamma$ vectors $s_{1},\ldots ,s_{H^{2}\cdot \gamma }\in N^{n}$ that satisfy the set of constraints listed below, represented by $E(d_{0},y_{1}y_{2}\cdots y_{\gamma })$,*

(15) $$\left\{\begin{array}{c} \left.\begin{array}{c} d_{0}\geq E^{-}\cdot s_{1}\\ d_{0}+E\cdot s_{1}\geq E^{-}\cdot s_{2}\\ Pr\left(d_{0}\right)=y_{1}\\ Pr\left(d_{0}+E\cdot s_{1}\right)=y_{1}\\ \cdots \\ d_{0}+E\cdot \sum_{i=1}^{H^{2}-1}s_{i}\geq E^{-}\cdot s_{H^{2}}\\ Pr\left(d_{0}+E\cdot \sum_{i=1}^{H^{2}}s_{i}\right)=y_{1} \end{array}\right\}\left(a\right)\\ \cdots \\ \left.\begin{array}{c} d_{0}+E\cdot \sum_{i=1}^{H^{2}\cdot \left(\gamma -1\right)}s_{i}\geq E^{-}\cdot s_{H^{2}\cdot \left(\gamma -1\right)+1}\\ Pr\left(d_{0}+E\cdot \sum_{i=1}^{H^{2}\cdot \left(\gamma -1\right)}s_{i}\right)=y_{\gamma }\\ \cdots \\ d_{0}+E\cdot \sum_{i=1}^{H^{2}\cdot \gamma -1}s_{i}\geq E^{-}\cdot s_{H^{2}\cdot \gamma }\\ Pr\left(d_{0}+E\cdot \sum_{i=1}^{H^{2}\cdot \gamma -1}s_{i}\right)=y_{\gamma }\\ Pr\left(d_{0}+E\cdot \sum_{i=1}^{H^{2}\cdot \gamma }s_{i}\right)=y_{\gamma }. \end{array}\right\}\left(b\right)\\ \left|\right|s_{i}{\left|\right|}_{1}=1,\;i=1,\ldots ,H^{2}\cdot \gamma \;\;\;\;\;\;\;\;\left(c\right) \end{array}\right.$$

**Proof of Lemma** **2.** (if) Since one state output corresponds to up to H system states, there are at most $H^{2}$ events between H states. By Lemma 1, if there exist $H^{2}$ vectors $u_{1},\ldots ,u_{H^{2}}$ satisfying constraints (15) (a), then at least one event sequence is generated from the initial state $d_{0}$ with the output $y_{1}$. Constraints (15) (b) have the same meaning as constraints (15) (a). Therefore, if there exist $H^{2}\cdot \gamma$ vectors $s_{1},\ldots ,s_{H^{2}\cdot \gamma }$ satisfying constraints $E(d_{0},y_{1}y_{2}\cdots y_{\gamma })$, an output sequence $y_{1}y_{2}\cdots y_{\gamma }$ is generated at the initial state $d_{0}$.

(only if) Let us suppose that an output sequence $y_{1}y_{2}\cdots y_{\gamma }$ is generated at the initial state $d_{0}$. According to Lemma 1, there exist $H^{2}\cdot \gamma$ vectors $s_{1},\ldots ,s_{H^{2}\cdot \gamma }$ corresponding to an event sequence yielding the output sequence $y_{1}y_{2}\cdots y_{\gamma }$ starting from the initial state $d_{0}$, such that constraints (15) (a), (b), and (c) are satisfied. □

**Theorem** **1.** *Consider a finite-state Vector DES $G=(D,\Sigma ,\xi ,d_{0},Y,Pr)$, $D\subseteq N^{n}$, and a fault $\varepsilon_{f}$. Let J be a positive integer such that $J\geq J_{min}$ (Implicitly, the value of J establishes the longest event sequence that can provide a generic state that enables $\varepsilon_{f}$. In [22], the minimum $J_{min}$ is presented to fully describe all states reachable from $d_{0}$ that enable $\varepsilon_{f}$, and these states are reached via a sequence not including $\varepsilon_{f}$. The system is supposed to be finite-state, which implies that the integer $J_{min}$ exists. An overestimation of $J_{min}$ [22] is given by |D|−1. Given an integer K that is positive, $\varepsilon_{f}$ is state-based K-diagnosable if and only if there exist $(\left(H^{2}+1\right)\cdot \left(J+K\right)+2H^{2})\in N$ vectors $u_{1},\ldots ,u_{J}$, $v_{1},\ldots ,v_{K}$,$s_{1},\ldots ,s_{H^{2}\cdot \left(J+K+2\right)}\in N^{n}$ such that*

$$\min_{s.t.D(d_{0},\varepsilon_{f},J,K)}\sum_{r=1}^{H^{2}\cdot \left(J+K+2\right)}s_{r}\left(\varepsilon_{f}\right)\neq 0,$$

*where the set of constraints $D(d_{0},\varepsilon_{f},J,K)$ is given in (16):*

(16) $$\left\{\begin{array}{c} \left.\begin{array}{c} d_{0}\geq E^{-}\cdot u_{1}\\ d_{0}+E\cdot u_{1}\geq E^{-}\cdot u_{2}\\ \cdots \\ d_{0}+E\cdot \sum_{i=1}^{J-1}u_{i}\geq E^{-}\cdot u_{J}\\ d_{0}+E\cdot \sum_{i=1}^{J}u_{i}\geq E^{-}\left(\varepsilon_{f}\right)\\ d_{0}+E\cdot \sum_{i=1}^{J}u_{i}+E\left(\cdot ,\varepsilon_{f}\right)\geq E^{-}\cdot v_{1}\\ d_{0}+E\cdot \sum_{i=1}^{J}u_{i}+E\left(\cdot ,\varepsilon_{f}\right)+E\cdot v_{1}\geq E^{-}\cdot v_{2}\\ \cdots \\ d_{0}+E\cdot \sum_{i=1}^{J}u_{i}+E\left(\cdot ,\varepsilon_{f}\right)+E\cdot \sum_{j=1}^{K-1}v_{j}\geq E^{-}\cdot v_{K}\\ \sum_{i=1}^{J}u_{i}\left(\varepsilon_{f}\right)=0\\ \left|\right|u_{i}{\left|\right|}_{1}=1,\;i=1,\ldots ,J\\ \left|\right|v_{j}{\left|\right|}_{1}=1,\;j=1,\ldots ,K\\ \left|\right|\sum_{j=1}^{K}v_{j}{\left|\right|}_{1}\geq K \end{array}\right\}\left(a\right)\\ \left.\begin{array}{c} E(d_{0},Pr\left(d_{0}\right)\cdots Pr\left(d_{0}+E\cdot \sum_{i=1}^{J}u_{i}+E\left(\cdot ,\varepsilon_{f}\right)+E\cdot \sum_{j=1}^{K}v_{j}\right) \end{array}\right\}\left(b\right) \end{array}\right.$$

**Proof of Theorem** **1.** (if) By $J\geq J_{min}$, the constraints (16) (a) describe all states reachable from $d_{0}$ that enable fault event $\varepsilon_{f}$. It follows that, if there exist $(\left(H^{2}+1\right)\cdot \left(J+K\right)+2H^{2})\in N$ vectors $u_{1},\ldots ,u_{J}$, $v_{1},\ldots ,v_{K}$,$s_{1},\ldots ,s_{H^{2}\cdot \left(J+K+2\right)}\in N^{n}$ fulfilling constraints (16) and

$$\min_{s.t.D(d_{0},\varepsilon_{f},J,K)}\sum_{r=1}^{H^{2}\cdot \left(J+K+2\right)}s_{r}\left(\varepsilon_{f}\right)\neq 0,$$

then every faulty event sequence $u\varepsilon_{f}v$ with $\varepsilon_{f}\notin u$ of an output sequence includes the fault event $\varepsilon_{f}$, such that there are at least K events in the postfix following the fault $\varepsilon_{f}$. Therefore, $\varepsilon_{f}$ is state-based K-diagnosable according to Definition 4.

(only if) Let us suppose that $\varepsilon_{f}$ is state-based K-diagnosable. According to Definition 4, for each state estimation $D_{e}$, which is provided after sufficient steps of state transition from $d_{fi}$, if $D_{e}\subseteq D_{fi}$, then the fault can be accurately identified in a limited delay.

Now, let us assume *ad absurdum* that

$$\min_{s.t.D(d_{0},\varepsilon_{f},J,K)}\sum_{r=1}^{H^{2}\cdot \left(J+K+2\right)}s_{r}\left(\varepsilon_{f}\right)=0.$$

Then, there should exist at least one output sequence and the corresponding event sequences that are $u\varepsilon_{f}v$ with $\varepsilon_{f}\notin u$ and $u^{\prime }v^{\prime }$ not containing $\varepsilon_{f}$, such that $\left|v|,\right|v^{\prime }|\geq K$. This implies that $\varepsilon_{f}$ is not state-based K-diagnosable, which contradicts that $\varepsilon_{f}$ is state-based K-diagnosable. □

The constraint (16) (b) will return the occurrence vectors of an event sequence generating the same output sequence with $u\varepsilon_{f}v$.

For a huge value of K, being state-based K-diagnosable could imply being practically undiagnosable. In practice, being state-based K-diagnosable for K≪|D| is essential when the number of states is high. Utilizing Theorem 1, the minimal K required for a diagnosable fault to be state-based K-diagnosable can be calculated by conducting a binary search on K, beginning at K=|D|/2.

### 4.2. Illustrative Example

**Figure 2:**
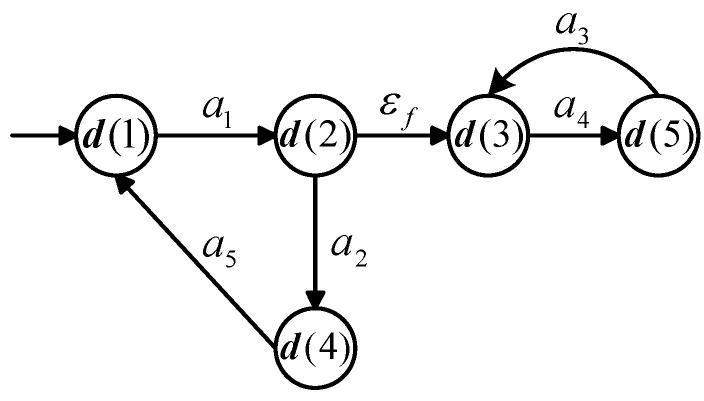
Vector DES model in Example 3.

**Example** **3.** *Consider a Vector DES **G** visualized in Figure 2 with alphabet $\Sigma =\{a_{1},a_{2},a_{3},a_{4},a_{5},\varepsilon_{f}\}$, where $\varepsilon_{f}$ represents the fault. The state vector d is defined as $d:={\left[d\left(1\right),d\left(2\right),d\left(3\right),d\left(4\right),d\left(5\right)\right]}^{T}\in N^{5\times 1}$. $d_{o}:=\left\{d\left(1\right),d\left(2\right),d\left(3\right),d\left(4\right)\right\}$, and $d_{uo}:=\left\{d\left(5\right)\right\}$. The initial state $d_{0}$ is ${\left[1,0,0,0,0\right]}^{T}$. Displacement matrix $E\in Z^{5\times 6}$ and $E^{-}\in Z^{5\times 6}$ are given as follows:*

$$\begin{array}{cc} & \begin{array}{cccccc} a_{1} & a_{2} & a_{3} & a_{4} & \varepsilon_{f} & a_{5} \end{array}\\ \begin{array}{c} d(1)\\ d(2)\\ d(3)\\ d(4)\\ d(5) \end{array} & \left(\begin{array}{cccccc} -1 & 0 & 0 & 0 & 0 & 1\\ 1 & -1 & 0 & 0 & -1 & 0\\ 0 & 0 & 1 & -1 & 1 & 0\\ 0 & 1 & 0 & 0 & 0 & -1\\ 0 & 0 & -1 & 1 & 0 & 0 \end{array}\right) \end{array}$$

$$\begin{array}{cc} & \begin{array}{cccccc} a_{1} & a_{2} & a_{3} & a_{4} & \varepsilon_{f} & a_{5} \end{array}\\ \begin{array}{c} d(1)\\ d(2)\\ d(3)\\ d(4)\\ d(5) \end{array} & \left(\begin{array}{cccccc} 1 & 0 & 0 & 0 & 0 & 0\\ 0 & 1 & 0 & 0 & 1 & 0\\ 0 & 0 & 0 & 1 & 0 & 0\\ 0 & 0 & 0 & 0 & 0 & 1\\ 0 & 0 & 1 & 0 & 0 & 0 \end{array}\right) \end{array}$$

*All states of* ***G*** *are listed in Table 2. In this system, $D_{n}=\left\{d_{0},d_{1},d_{3}\right\}$ and $D_{f}=\left\{d_{2},d_{4}\right\}$. We solve an ILP problem and a full state enumeration is avoided. The |D| is calculated using Netlab software 1.75 and it equals 5. We set J=5 and H=2. If we select K=1, then*

$$\min_{s.t.D(d_{0},\varepsilon_{f},5,1)}\sum_{r=1}^{32}s_{r}\left(\varepsilon_{f}\right)\neq 0,$$

*where the set of constraints $D(d_{0},\varepsilon_{f},5,1)$ is given in (17):*

(17) $$\left\{\begin{array}{c} \left.\begin{array}{c} d_{0}\geq E^{-}\cdot u_{1}\\ d_{0}+E\cdot u_{1}\geq E^{-}\cdot u_{2}\\ \cdots \\ d_{0}+E\cdot \sum_{i=1}^{5}u_{i}\geq E^{-}\left(\varepsilon_{f}\right)\\ d_{0}+E\cdot \sum_{i=1}^{5}u_{i}+E\left(\cdot ,\varepsilon_{f}\right)\geq E^{-}\cdot v_{1}\\ \sum_{i=1}^{5}u_{i}\left(\varepsilon_{f}\right)=0\\ \left|\right|u_{i}{\left|\right|}_{1}=1,\;i=1,\ldots ,5\\ \left|\right|v_{j}{\left|\right|}_{1}=1,\;j=1\\ \left|\right|\sum_{j=1}^{1}v_{j}{\left|\right|}_{1}\geq 1 \end{array}\right\}\left(a\right)\\ \left.\begin{array}{c} E(d_{0},Pr\left(d_{0}\right)\cdots Pr\left(d_{0}+E\cdot \sum_{i=1}^{5}u_{i}+E\left(\cdot ,\varepsilon_{f}\right)+E\cdot v_{1}\right). \end{array}\right\}\left(b\right) \end{array}\right.$$

*If we select K=2, then*

$$\min_{s.t.D(d_{0},\varepsilon_{f},5,2)}\sum_{r=1}^{36}s_{r}\left(\varepsilon_{f}\right)\neq 0.$$

*Therefore, $\varepsilon_{f}$ is state-based 1-diagnosable. We have validated this result by employing the YALMIP software tool R20180612 [39] to solve the ILP problem.*

### 4.3. Complexity Analysis

The primary computational burden of the proposed method lies in solving Equation (16), which can be reformulated as an ILP model. It is widely acknowledged that ILP problems are generally NP-hard. Notably, the computational complexity of solving an ILP problem is heavily influenced by the number of constraints and variables involved. Assuming that J is provided, it is easy to deduce that (16) (a) contains (J+K)·|Σ| variables, while (16) (a) contains (2J+2K+2)·n+1 constraints. The number of variables in (16) (b) is $H^{2}\cdot \left(J+K+2\right)\cdot \left|\Sigma \right|$, while that of constraints in (16) (b) is $H^{2}\cdot \left(2J+2K+2\right)$. The total number of variables in (16) is $\left(J+K+H^{2}\cdot \left(J+K+2\right))\cdot |\Sigma \right|$, while the total number of constraints is $2\left(J+K+1\right)\cdot \left(H^{2}+n\right)+1$. Furthermore, if J and K are given, regardless of the initial state, the numbers of constraints and variables rise linearly with the size of a Vector DES.

## 5. State-Based Fault Online Diagnosis

### 5.1. State-Based Fault Online Diagnosis of Finite-State Vector DESs

For online diagnosis, the current condition of a system is determined by the recorded state output. We do not rely on a diagnoser-based approach to achieve online diagnosis. The following predicates are presented to divide the outputs of current states based on the condition of a system.

**Definition** **5** (Faulty predicate $P_{f}$)**.** *Given a Vector DES $G=(D,\Sigma ,\xi ,d_{0},Y,Pr)$, $D\subseteq N^{n}$, and a fault $\varepsilon_{f}$, the faulty predicate $P_{f}$ with regard to **G** identifies a state subset $D_{P_{f}}\subseteq D_{o}$ by defining*

$$D_{P_{f}}:=\left\{d_{o}\in D_{o}|\forall s\in L\left(G,d_{0}\right):Pr\left(\xi \left(d_{0},s\right)\right)=d_{o}\wedge v_{\varepsilon_{f}}\left(s\right)\neq 0\right\},$$

*or equivalently,*

$$d_{o}⊧P_{f}\;\;\mathrm{iff}\;\;\forall s\in L\left(G,d_{0}\right):Pr\left(\xi \left(d_{0},s\right)\right)=d_{o}\wedge v_{\varepsilon_{f}}\left(s\right)\neq 0.$$

The following provides an illustration of the definition of faulty predicate $P_{f}$. Given a state output satisfying $P_{f}$, all states in the set of estimation are reachable via the sequences containing a fault, and the current condition of G is faulty.

**Definition** **6** (Normal predicate $P_{n}$)**.** *Given a Vector DES $G=(D,\Sigma ,\xi ,d_{0},Y,Pr)$, $D\subseteq N^{n}$, and a fault $\varepsilon_{f}$, the normal predicate $P_{n}$ with regard to **G** identifies a state subset $D_{P_{n}}\subseteq D_{o}$ by defining*

$$D_{P_{n}}:=\left\{d_{o}\in D_{o}|\forall s\in L\left(G,d_{0}\right):Pr\left(\xi \left(d_{0},s\right)\right)=d_{o}\wedge v_{\varepsilon_{f}}\left(s\right)=0\right\},$$

*or equivalently,*

$$d_{o}⊧P_{n}\;\;\mathrm{iff}\;\;\forall s\in L\left(G,d_{0}\right):Pr\left(\xi \left(d_{0},s\right)\right)=d_{o}\wedge v_{\varepsilon_{f}}\left(s\right)=0.$$

The following provides an illustration of the definition of normal predicate $P_{n}$. Given a state output satisfying $P_{n}$, all states in the set of estimation are reachable via the sequences not containing a fault, and the current condition of G is normal.

**Definition** **7** (Uncertain predicate $P_{un}$)**.** *Given a Vector DES $G=(D,\Sigma ,\xi ,d_{0},Y,Pr)$, $D\subseteq N^{n}$, and a fault $\varepsilon_{f}$, the uncertain predicate $P_{un}$ with regard to **G** identifies a state subset $D_{P_{un}}\subseteq D_{o}$ by defining*

$D_{P_{un}}:=\left\{d_{o}\in D_{o}|\exists s,s^{\prime }\in L\left(G,d_{0}\right):Pr\left(\xi \left(d_{0},s\right)\right)=d_{o}\wedge Pr\left(\xi \left(d_{0},s^{\prime }\right)\right)=d_{o}\wedge v_{\varepsilon_{f}}\left(s\right)=0\wedge v_{\varepsilon_{f}}\left(s^{\prime }\right)\neq 0\right\},$

*or equivalently,*

$d_{o}⊧P_{un}\;\;\mathrm{iff}\;\;\exists s,s^{\prime }\in L\left(G,d_{0}\right):Pr\left(\xi \left(d_{0},s\right)\right)=d_{o}\wedge Pr\left(\xi \left(d_{0},s^{\prime }\right)\right)=d_{o}\wedge v_{\varepsilon_{f}}\left(s\right)=0\wedge v_{\varepsilon_{f}}\left(s^{\prime }\right)\neq 0.$

For any state output $d_{o}⊧P_{un}$, there exist at least two states $d,d^{\prime }\in D$ with the identical observation, such that d is reachable via a sequence containing a fault, and $d^{\prime }$ is reachable via a sequence not containing a fault. The current condition of G is uncertain.

**Example** **4.** *For Example 3, if ${\left[0,1,0,0\right]}^{T}$ is observed, the corresponding state estimation set is $\left\{{\left[0,1,0,0,1\right]}^{T}\right\}$. Each state in this set is reachable via the sequences containing a fault and ${\left[0,1,0,0\right]}^{T}$ satisfies $P_{f}$, implying that a fault occurs.*

**Proposition** **1.** *Given a finite-state Vector DES $G=(D,\Sigma ,\xi ,d_{0},Y,Pr)$, $D\subseteq N^{n}$, a fault $\varepsilon_{f}$, and the current state output $d_{c}\in D_{o}$, $d_{c}⊧P_{f}$ if and only if there exist ρ vectors $u_{1},\ldots ,u_{\rho }\in N^{n}$, such that*

$$\mathrm{ILPP}\;1:\min_{s.t.F(d_{0},\rho ,d_{c})}\sum_{i=1}^{\rho }u_{i}\left(\varepsilon_{f}\right)\neq 0,$$

*where the set of constraints $F(d_{0},\rho ,d_{c})$ is given in (18):*

(18) $$\left\{\begin{array}{c} \left.\begin{array}{c} d_{0}\geq E^{-}\cdot u_{1}\\ d_{0}+E\cdot u_{1}\geq E^{-}\cdot u_{2}\\ \cdots \\ d_{0}+E\cdot \sum_{i=1}^{\rho -1}u_{i}\geq E^{-}\cdot u_{\rho } \end{array}\right\}\left(a\right)\\ \left.\begin{array}{c} Pr\left(d_{0}+E\cdot \sum_{i=1}^{\rho }u_{i}\right)=d_{c}. \end{array}\right\}\left(b\right) \end{array}\right.$$

**Proof of Proposition** **1.** (if) By Lemma 1, if there exist ρ vectors $u_{1},\ldots ,u_{\rho }$ satisfying constraints (18) (a), then at least one event sequence is generated from the initial state $d_{0}$. Suppose that there exist ρ vectors $u_{1},\ldots ,u_{\rho }$, such that $\min_{s.t.F(d_{0},\rho ,d_{c})}\sum_{i=1}^{\rho }u_{i}\left(\varepsilon_{f}\right)\neq 0$, which implies that any state d in the state estimation set of $d_{c}$ according to the constraint (18) (b) is reachable via fault $\varepsilon_{f}$. By Definition 5, $d_{c}⊧P_{f}$ and the fault occurs.

(only if) Assume $d_{c}⊧P_{f}$. It follows that, by Definition 5, all states in the set of estimation of $d_{c}$ are reachable via sequences containing a fault.

Now, let us assume *ad absurdum* that

$$\min_{s.t.F(d_{0},\rho ,d_{c})}\sum_{i=1}^{\rho }u_{i}\left(\varepsilon_{f}\right)=0.$$

Then, there should exist at least one state d in the state estimation set of $d_{c}$ that is reachable not via fault $\varepsilon_{f}$. This implies that $d_{c}$ does not satisfy $P_{f}$, which contradicts that $d_{c}⊧P_{f}$. □

**Proposition** **2.** *Given a finite-state Vector DES $G=(D,\Sigma ,\xi ,d_{0},Y,Pr)$, $D\subseteq N^{n}$, a fault $\varepsilon_{f}$, and the current state output $d_{c}\in D_{o}$, $d_{c}⊧P_{n}$ if and only if there exist ρ vectors $u_{1},\ldots ,u_{\rho }\in N^{n}$, such that*

$$\mathrm{ILPP}\;2:\max_{s.t.N(d_{0},\rho ,d_{c})}\sum_{i=1}^{\rho }u_{i}\left(\varepsilon_{f}\right)=0,$$

*where the set of constraints $N(d_{0},\rho ,d_{c})$ is presented in (19):*

(19) $$\left\{\begin{array}{c} \left.\begin{array}{c} d_{0}\geq E^{-}\cdot u_{1}\\ d_{0}+E\cdot u_{1}\geq E^{-}\cdot u_{2}\\ \cdots \\ d_{0}+E\cdot \sum_{i=1}^{\rho -1}u_{i}\geq E^{-}\cdot u_{\rho } \end{array}\right\}\left(a\right)\\ \left.\begin{array}{c} Pr\left(d_{0}+E\cdot \sum_{i=1}^{\rho }u_{i}\right)=d_{c}. \end{array}\right\}\left(b\right) \end{array}\right.$$

**Proof of Proposition** **2.** (if) By Lemma 1, if there exist ρ vectors $u_{1},\ldots ,u_{\rho }$ satisfying constraints (19) (a), then at least one event sequence is generated from the initial state $d_{0}$. Suppose that there exist ρ vectors $u_{1},\ldots ,u_{\rho }$, such that $\max_{s.t.N(d_{0},\rho ,d_{c})}\sum_{i=1}^{\rho }u_{i}\left(\varepsilon_{f}\right)=0$, which implies that any state d in the state estimation set of $d_{c}$ according to the constraint (19) (b) is reachable not via fault $\varepsilon_{f}$. By Definition 6, $d_{c}⊧P_{n}$ and the fault does not occur.

(only if) Assume $d_{c}⊧P_{n}$. It follows that, by Definition 6, all states in the set of estimation of $d_{c}$ are reachable via sequences not containing a fault.

Now, let us assume *ad absurdum* that

$$\max_{s.t.N(d_{0},\rho ,d_{c})}\sum_{i=1}^{\rho }u_{i}\left(\varepsilon_{f}\right)\neq 0.$$

Then, there should exist at least one state d in the state estimation set of $d_{c}$ that is reachable via fault $\varepsilon_{f}$. This implies that $d_{c}$ does not satisfy $P_{n}$, which contradicts that $d_{c}⊧P_{n}$. □

**Proposition** **3.** *Given a finite-state Vector DES $G=(D,\Sigma ,\xi ,d_{0},Y,Pr)$, $D\subseteq N^{n}$, a fault $\varepsilon_{f}$, and the current state output $d_{c}\in D_{o}$, $d_{c}⊧P_{un}$ if and only if there exist ρ+λ vectors $u_{1},\ldots ,u_{\rho }$, $v_{1},\ldots ,v_{\lambda }\in N^{n}$ such that*

$$\mathrm{ILPP}\;3:\min_{s.t.U(d_{0},\lambda ,\rho ,d_{c})}\sum_{j=1}^{\lambda }v_{j}\left(\varepsilon_{f}\right)=0,$$

*where the set of constraints $U(d_{0},\lambda ,\rho ,d_{c})$ is given in (20):*

(20) $$\left\{\begin{array}{c} \left.\begin{array}{c} d_{0}\geq E^{-}\cdot u_{1}\\ d_{0}+E\cdot u_{1}\geq E^{-}\cdot u_{2}\\ \cdots \\ d_{0}+E\cdot \sum_{i=1}^{\rho -1}u_{i}\geq E^{-}\cdot u_{\rho } \end{array}\right\}\left(a\right)\\ \left.\begin{array}{c} d_{0}\geq E^{-}\cdot v_{1}\\ d_{0}+E\cdot v_{1}\geq E^{-}\cdot v_{2}\\ \cdots \\ d_{0}+E\cdot \sum_{j=1}^{\lambda -1}v_{j}\geq E^{-}\cdot v_{\lambda } \end{array}\right\}\left(b\right)\\ \left.\begin{array}{c} \sum_{i=1}^{\rho }u_{i}\left(\varepsilon_{f}\right)\neq 0 \end{array}\right\}\left(c\right)\\ \left.\begin{array}{c} Pr\left(d_{0}+E\cdot \sum_{i=1}^{\rho }u_{i}\right)=d_{c}\\ Pr\left(d_{0}+E\cdot \sum_{j=1}^{\lambda }v_{j}\right)=d_{c}. \end{array}\right\}\left(d\right) \end{array}\right.$$

**Proof of Proposition** **3.** (if) By Lemma 1, if there exist ρ+λ vectors $u_{1},\ldots ,u_{\rho }$, $v_{1},\ldots ,v_{\lambda }$ satisfying constraints (20) (a) and (b), then at least one event sequence is generated from the initial state $d_{0}$. Suppose that there exist ρ+λ vectors $u_{1},\ldots ,u_{\rho }$, $v_{1},\ldots ,v_{\lambda }\in N^{n}$ such that $\min_{s.t.U(d_{0},\lambda ,\rho ,d_{c})}\sum_{j=1}^{\lambda }v_{j}\left(\varepsilon_{f}\right)=0$. Then, there exist two states with the same state output $d_{c}$ according to constraints (20) (d), for which one state is not generated via a fault but another is generated via at least one fault according to the constraint (20) (c). By Definition 7, $d_{c}⊧P_{un}$ and the system condition is uncertain.

(only if) Assume $d_{c}⊧P_{un}$. It follows that, by Definition 7, for any state d in the state estimation set of $d_{c}$, there exists another state $d^{\prime }$ such that these two states have the same output $d_{c}$. However, d is reachable via a sequence containing a fault, and $d^{\prime }$ is reachable via a sequence not containing a fault.

Now, let us assume *ad absurdum* that

$$\min_{s.t.U(d_{0},\lambda ,\rho ,d_{c})}\sum_{j=1}^{\lambda }v_{j}\left(\varepsilon_{f}\right)\neq 0.$$

Then, each state in the state estimation set of $d_{c}$ is reachable via fault $\varepsilon_{f}$. This implies that $d_{c}$ does not satisfy $P_{un}$, which contradicts that $d_{c}⊧P_{un}$. □

**Proposition** **4.** *Given a finite-state Vector DES $G=(D,\Sigma ,\xi ,d_{0},Y,Pr)$, $D\subseteq N^{n}$, and a fault $\varepsilon_{f}$, the upper bounds of ρ and λ satisfy*

(21) $$\rho ,\lambda \leq \|\sum_{h\in T(G)}{h\|}_{1}+\left(|D|-1\right).$$

**Proof of Proposition** **4.** Denote by $\sigma =\sigma_{1}\sigma_{2}\ldots \sigma_{k}$ the longest sequence. In a finite-state Vector DES G, the length of σ can be arbitrarily long. Here, we just need to compare the projections of states. The states of G are finite, whose number is |D|. The farthest state can be reached from $d_{0}$ via σ by the occurrence of the maximum number of events without reaching two times the same intermediate state. $\|\sum_{h\in T(G)}{h\|}_{1}$ covers all minimal support E-invariants. Therefore, the length of σ is less than $\left(\right\|\sum_{h\in T(G)}{h\|}_{1}+\left(|D\right|-1))$, i.e., ρ and λ are less than $\left(\right\|\sum_{h\in T(G)}{h\|}_{1}+\left(|D\right|-1))$. □

Now, an online procedure for Problem 2 is ready to be developed. The basic procedures for performing state-based diagnosis in a finite-state Vector DES using the ILP approach only are outlined in Algorithm 1, where is guaranteed to be correct by Propositions 1–4.
**Algorithm 1:** State-based online fault diagnosis of a finite-state Vector DES
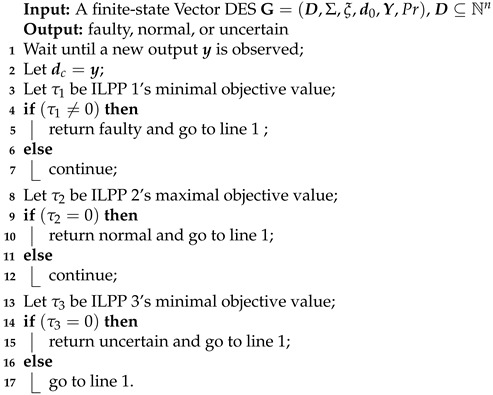


### 5.2. Illustrative Example

**Example** **5.** *Consider the Vector DES **G** presented in Example 3. The variable |D| is calculated with Netlab software 1.75 and it equals 5. Then, the minimal support E-invariants are calculated with Netlab: $h_{1}={\left[1,1,0,0,0,1\right]}^{T}$, $h_{2}={\left[0,0,1,1,0,0\right]}^{T}$.*

*Proposition 4 gives the upper bounds of ρ and λ, i.e.,*

$\rho ,\lambda \leq \|h_{1}+h_{2}{\|}_{1}+\left(|D|-1\right)=5+4=9.$

*Before applying Algorithm 1 to the Vector DES **G**, the corresponding ILPPs 1, 2, and 3 are obtained as follows.*

$\mathrm{ILPP}\;1:\min_{s.t.F(d_{0},9,d_{c})}\sum_{i=1}^{9}u_{i}\left(\varepsilon_{f}\right)\neq 0,$

*where the set of constraints $F(d_{0},9,d_{c})$ is given in (22):*

(22) $$\left\{\begin{array}{c} \left.\begin{array}{c} d_{0}\geq E^{-}\cdot u_{1}\\ d_{0}+E\cdot u_{1}\geq E^{-}\cdot u_{2}\\ \cdots \\ d_{0}+E\cdot \sum_{i=1}^{8}u_{i}\geq E^{-}\cdot u_{9} \end{array}\right\}\left(a\right)\\ \left.\begin{array}{c} Pr\left(d_{0}+E\cdot \sum_{i=1}^{9}u_{i}\right)=d_{c}. \end{array}\right\}\left(b\right) \end{array}\right.$$

$\mathrm{ILPP}\;2:\max_{s.t.N(d_{0},9,d_{c})}\sum_{i=1}^{9}u_{i}\left(\varepsilon_{f}\right)=0,$

*where the set of constraints $N(d_{0},9,d_{c})$ is presented in (23):*

(23) $$\left\{\begin{array}{c} \left.\begin{array}{c} d_{0}\geq E^{-}\cdot u_{1}\\ d_{0}+E\cdot u_{1}\geq E^{-}\cdot u_{2}\\ \cdots \\ d_{0}+E\cdot \sum_{i=1}^{8}u_{i}\geq E^{-}\cdot u_{9} \end{array}\right\}\left(a\right)\\ \left.\begin{array}{c} Pr\left(d_{0}+E\cdot \sum_{i=1}^{9}u_{i}\right)=d_{c}. \end{array}\right\}\left(b\right) \end{array}\right.$$

$\mathrm{ILPP}\;3:\min_{s.t.U(d_{0},9,9,d_{c})}\sum_{j=1}^{9}v_{j}\left(\varepsilon_{f}\right)=0,$

*where the set of constraints $U(d_{0},9,9,d_{c})$ is given in (24):*

(24) $$\left\{\begin{array}{c} \left.\begin{array}{c} d_{0}\geq E^{-}\cdot u_{1}\\ d_{0}+E\cdot u_{1}\geq E^{-}\cdot u_{2}\\ \cdots \\ d_{0}+E\cdot \sum_{i=1}^{8}u_{i}\geq E^{-}\cdot u_{9} \end{array}\right\}\left(a\right)\\ \left.\begin{array}{c} d_{0}\geq E^{-}\cdot v_{1}\\ d_{0}+E\cdot v_{1}\geq E^{-}\cdot v_{2}\\ \cdots \\ d_{0}+E\cdot \sum_{j=1}^{8}v_{j}\geq E^{-}\cdot v_{9} \end{array}\right\}\left(b\right)\\ \left.\begin{array}{c} \sum_{i=1}^{9}u_{i}\left(\varepsilon_{f}\right)\neq 0 \end{array}\right\}\left(c\right)\\ \left.\begin{array}{c} Pr\left(d_{0}+E\cdot \sum_{i=1}^{9}u_{i}\right)=d_{c}\\ Pr\left(d_{0}+E\cdot \sum_{j=1}^{9}v_{j}\right)=d_{c}. \end{array}\right\}\left(d\right) \end{array}\right.$$

*When $d_{c}=y={\left[0,1,0,0\right]}^{T}$ is observed, the output of Algorithm 1 is “normal”. When $d_{c}=y={\left[0,0,1,0\right]}^{T}$ is observed, the output of Algorithm 1 is “faulty”.*

### 5.3. Complexity Analysis

The solutions to ILPPs 1, 2, and 3 are the primary source of Algorithm 1’s computational expense. An ILP problem is believed to be NP-hard in general. Let us assume that ρ and λ are given; it is easy to deduce that ILPP 1 contains ρ·|Σ| variables, while ILPP 1 contains (ρ+1)·n constraints. ILPP 2 contains ρ·|Σ| variables, while ILPP 2 contains (ρ+1)·n constraints. ILPP 3 contains (ρ+λ)·|Σ| variables, while ILPP 3 contains (ρ+λ+3)·n constraints.

## 6. Example: A Production Line Repairing Damaged Parts

**Figure 3:**
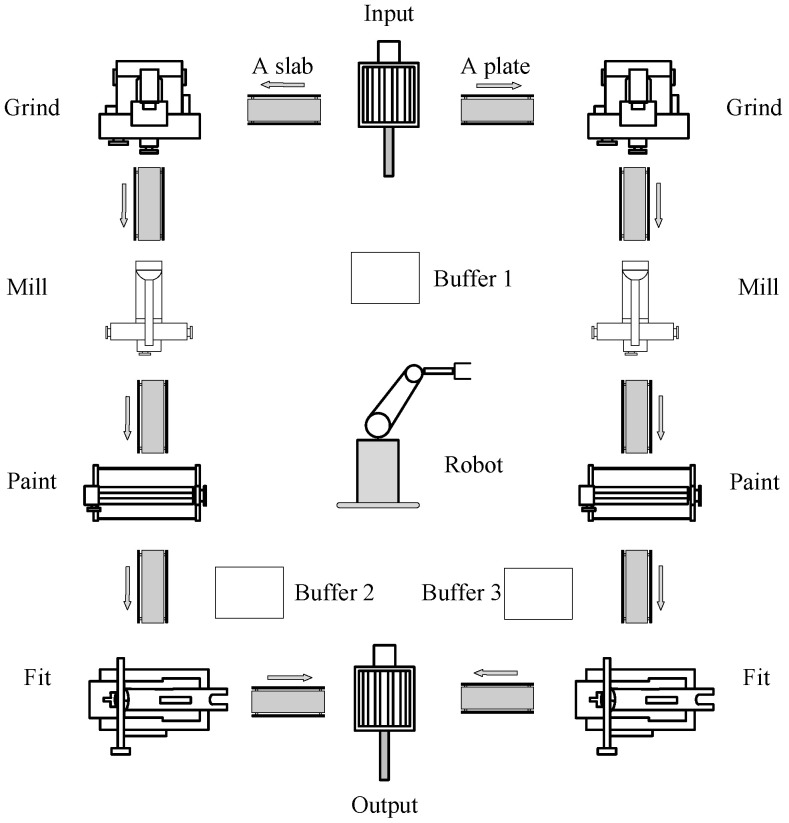
Schematic of the production line repairing damaged parts.

Consider a production line repairing damaged parts as shown in Figure 3, namely metallic slabs where a plate and a slab have been placed in decentralized positions. The production line consists of two lines, and each line consists of four parts, i.e., a numerically controlled grinding machine, a numerically controlled milling machine, a painting machine, and a numerically controlled fitter. Slabs and plates are separated when a damaged metallic slab is ready for repair, with the slab going in the left line and the plate in the right. Parts are fixed in the two lines consisting of ground, milled, painting, and fitted. Ultimately, the robot correctly positions a single metallic plate in the slab. The whole process is repeated. A new damaged metallic slab enters the production line as input. Slabs and plates are separated when a new damaged metallic slab is ready for repair, with the slab going in the left line and the plate in the right.

**Figure 4:**
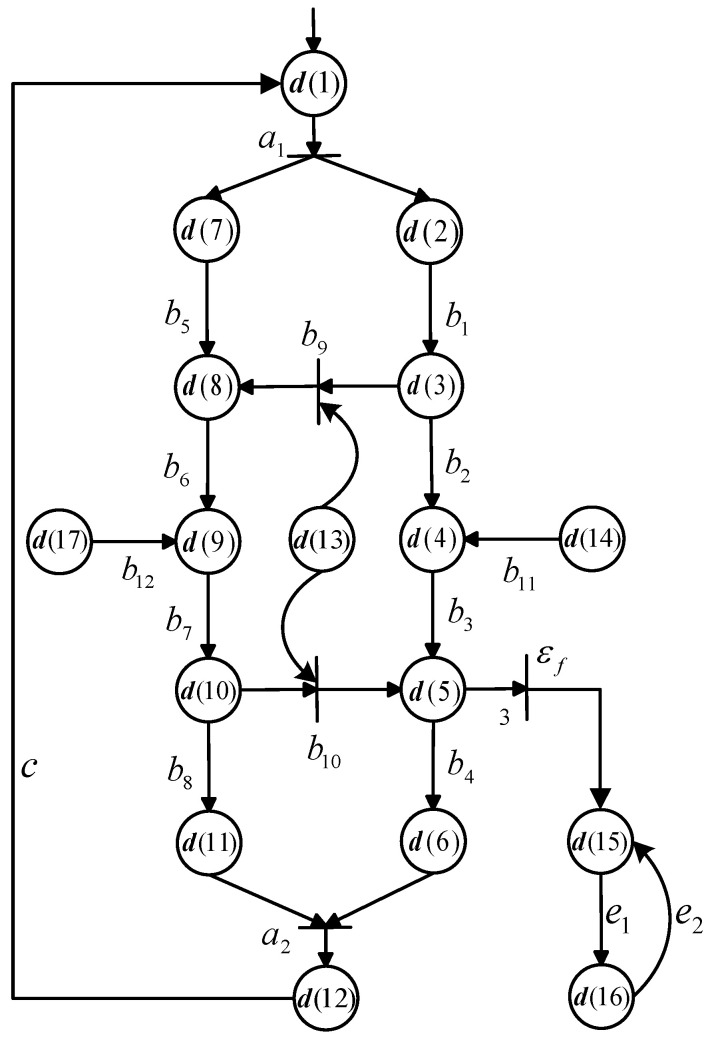
A Vector DES model of the production line repairing damaged parts in Figure 3.

The Vector DES model of the production line is visualized in Figure 4, where the meanings of events and state components are shown in Table 3 and Table 4, respectively. We define the state vector d as d:=[d(1),d(2),d(3),d(4),d(5), d(6), d(7), d(8), d(9), d(10), d(11), d(12), d(13), ${d\left(14\right),d\left(15\right),d\left(16\right),d\left(17\right)]}^{T}\in N^{17\times 1}$, $d_{o}:=\{d\left(1\right),d\left(2\right),d\left(3\right),d\left(4\right),d\left(5\right),d\left(6\right),d\left(7\right)$, d(8), d(9),d(10),d(11),d(12),d(13), d(14), d(15), d(17)}, and $d_{uo}:=\left\{d\left(16\right)\right\}$. $\Sigma =\{a_{1}$, $a_{2}$, $b_{1}$, $b_{2}$, $b_{3}$, $b_{4}$, $b_{5}$, $b_{6}$, $b_{7}$, $b_{8}$, $b_{9}$, $b_{10}$, $b_{11}$, $b_{12}$, *c*, $e_{1}$, $e_{2}$, $\varepsilon_{f}\}$, where $\varepsilon_{f}$ represents the fault. The initial state $d_{0}$ is [1,0,0,0,0,0,0, 0, 0, 0, 0, 0, 1, 1, 0, 0, ${1]}^{T}$.

The Vector DES model of the production line is finite-state and has a total of 1149 states, which has been successfully verified by Netlab. We set J=1148 and H=2. If we select K=1, then

$$\min_{s.t.D(d_{0},\varepsilon_{f},1148,1)}\sum_{r=1}^{4604}s_{r}\left(\varepsilon_{f}\right)=1\neq 0,$$

where the set of constraints $D(d_{0},\varepsilon_{f},1148,1)$ is given in (25):

(25) $$\left\{\begin{array}{c} \left.\begin{array}{c} d_{0}\geq E^{-}\cdot u_{1}\\ d_{0}+E\cdot u_{1}\geq E^{-}\cdot u_{2}\\ \cdots \\ d_{0}+E\cdot \sum_{i=1}^{1148}u_{i}\geq E^{-}\left(\varepsilon_{f}\right)\\ d_{0}+E\cdot \sum_{i=1}^{1148}u_{i}+E\left(\cdot ,\varepsilon_{f}\right)\geq E^{-}\cdot v_{1}\\ \sum_{i=1}^{1148}u_{i}\left(\varepsilon_{f}\right)=0\\ \left|\right|u_{i}{\left|\right|}_{1}=1,\;i=1,\ldots ,1148\\ \left|\right|v_{j}{\left|\right|}_{1}=1,\;j=1\\ \left|\right|\sum_{j=1}^{1}v_{j}{\left|\right|}_{1}\geq 1 \end{array}\right\}\left(a\right)\\ \left.\begin{array}{c} E(d_{0},Pr\left(d_{0}\right)\cdots Pr\left(d_{0}+E\cdot \sum_{i=1}^{1148}u_{i}+E\left(\cdot ,\varepsilon_{f}\right)+E\cdot v_{1}\right). \end{array}\right\}\left(b\right) \end{array}\right.$$

Therefore, $\varepsilon_{f}$ is state-based 1-diagnosable.

Then, the minimal support E-invariants are calculated with Netlab:

$h_{1}={\left[1,1,1,1,1,1,1,1,1,0,0,0,1,0,1,0,0\right]}^{T}$,

$h_{2}={\left[0,0,0,0,0,0,0,0,0,0,0,0,0,0,0,1,1\right]}^{T}$.

Proposition 4 gives the upper bounds of ρ and λ, i.e.,

$\rho ,\lambda \leq \|h_{1}+h_{2}{\|}_{1}+\left(|D|-1\right)=1161.$

Before applying Algorithm 1 to the Vector DES model of the production line, the corresponding ILPPs 1, 2, and 3 are obtained as follows.

$\mathrm{ILPP}\;1:\min_{s.t.F(d_{0},1161,d_{c})}\sum_{i=1}^{1161}u_{i}\left(\varepsilon_{f}\right)\neq 0,$

where the set of constraints $F(d_{0},1161,d_{c})$ is given in (26):

(26) $$\left\{\begin{array}{c} \left.\begin{array}{c} d_{0}\geq E^{-}\cdot u_{1}\\ d_{0}+E\cdot u_{1}\geq E^{-}\cdot u_{2}\\ \cdots \\ d_{0}+E\cdot \sum_{i=1}^{1160}u_{i}\geq E^{-}\cdot u_{1161} \end{array}\right\}\left(a\right)\\ \left.\begin{array}{c} Pr\left(d_{0}+E\cdot \sum_{i=1}^{1161}u_{i}\right)=d_{c}. \end{array}\right\}\left(b\right) \end{array}\right.$$

$\mathrm{ILPP}\;2:\max_{s.t.N(d_{0},1161,d_{c})}\sum_{i=1}^{1161}u_{i}\left(\varepsilon_{f}\right)=0,$

where the set of constraints $N(d_{0},1161,d_{c})$ is presented in (27):

(27) $$\left\{\begin{array}{c} \left.\begin{array}{c} d_{0}\geq E^{-}\cdot u_{1}\\ d_{0}+E\cdot u_{1}\geq E^{-}\cdot u_{2}\\ \cdots \\ d_{0}+E\cdot \sum_{i=1}^{1160}u_{i}\geq E^{-}\cdot u_{1161} \end{array}\right\}\left(a\right)\\ \left.\begin{array}{c} Pr\left(d_{0}+E\cdot \sum_{i=1}^{1161}u_{i}\right)=d_{c}. \end{array}\right\}\left(b\right) \end{array}\right.$$

$\mathrm{ILPP}\;3:\min_{s.t.U(d_{0},1161,1161,d_{c})}\sum_{j=1}^{1161}v_{j}\left(\varepsilon_{f}\right)=0,$

where the set of constraints $U(d_{0},1161,1161,d_{c})$ is given in (28):

(28) $$\left\{\begin{array}{c} \left.\begin{array}{c} d_{0}\geq E^{-}\cdot u_{1}\\ d_{0}+E\cdot u_{1}\geq E^{-}\cdot u_{2}\\ \cdots \\ d_{0}+E\cdot \sum_{i=1}^{1160}u_{i}\geq E^{-}\cdot u_{1161} \end{array}\right\}\left(a\right)\\ \left.\begin{array}{c} d_{0}\geq E^{-}\cdot v_{1}\\ d_{0}+E\cdot v_{1}\geq E^{-}\cdot v_{2}\\ \cdots \\ d_{0}+E\cdot \sum_{j=1}^{1160}v_{j}\geq E^{-}\cdot v_{1161} \end{array}\right\}\left(b\right)\\ \left.\begin{array}{c} \sum_{i=1}^{1161}u_{i}\left(\varepsilon_{f}\right)\neq 0 \end{array}\right\}\left(c\right)\\ \left.\begin{array}{c} Pr\left(d_{0}+E\cdot \sum_{i=1}^{1161}u_{i}\right)=d_{c}\\ Pr\left(d_{0}+E\cdot \sum_{j=1}^{1161}v_{j}\right)=d_{c}. \end{array}\right\}\left(d\right) \end{array}\right.$$

When $d_{c}=y={\left[0,1,0,0,0,0,1,0,0,0,0,0,1,1,0\right]}^{T}$ is observed, the output of Algorithm 1 is “normal”. We have validated these results by employing the YALMIP software tool to solve the ILP problems.

## 7. Conclusions

This paper addresses a state-based approach to solving the problems of the verification of K-diagnosability and online diagnosis of a Vector DES by formulating and solving ILP problems. A necessary and sufficient condition for verifying K-diagnosability of a finite-state Vector DES is presented. Several ILP problems are built according to a state output. The online diagnosis result is obtained by giving distinct objective functions to the ILP problem. The proposed approach requires neither any search of paths in graphs nor any estimation of the reachability set. By changing the structure of input of the Vector DES to the suggested ILP problems, it can therefore be applied to other Vector DESs. In future work, we will optimize parameters $J_{min}$, λ, and ρ of the ILP problems to make the procedure more efficient.

## Figures and Tables

**Table 1:** All states of Vector DES **G** in Example 1.

State	State Vector
$d_{0}$	${\left[1,0,0,0\right]}^{T}$
$d_{1}$	${\left[0,1,0,0\right]}^{T}$
$d_{2}$	${\left[0,0,1,0\right]}^{T}$
$d_{3}$	${\left[0,0,0,1\right]}^{T}$

**Table 2:** All states of Vector DES **G** in Example 3.

State	State Vector
$d_{0}$	${\left[1,0,0,0,0\right]}^{T}$
$d_{1}$	${\left[0,1,0,0,0\right]}^{T}$
$d_{2}$	${\left[0,0,1,0,0\right]}^{T}$
$d_{3}$	${\left[0,0,0,1,0\right]}^{T}$
$d_{4}$	${\left[0,0,0,0,1\right]}^{T}$

**Table 3:** Event of Figure 4.

Event	Meaning	Fault
$a_{1}$	*A damaged part is separated*	*No*
$a_{2}$	*A metallic plate is inserted in the slab*	*No*
$b_{1}$	*Right line is grinding*	*No*
$b_{2}$	*Right line is milling*	*No*
$b_{3}$	*Right line is painting*	*No*
$b_{4}$	*Right line is fitting*	*No*
$b_{5}$	*Left line is grinding*	*No*
$b_{6}$	*Left line is milling*	*No*
$b_{7}$	*Left line is painting*	*No*
$b_{8}$	*Left line is fitting*	*No*
$b_{9}$	*Load a supplementary slab from Buffer 1*	*No*
$b_{10}$	*Load a supplementary plate from Buffer 1*	*No*
$b_{11}$	*Load a supplementary plate from Buffer 3*	*No*
$b_{12}$	*Load a supplementary slab from Buffer 2*	*No*
*c*	*Repeat the whole process*	*No*
$e_{1}$	*Self-replenish pigment*	*No*
$e_{2}$	*Pigment deficiency response*	*No*
$\varepsilon_{f}$	*There is not enough pigment to paint*	*Yes*

**Table 4:** State component of Figure 4.

State Component	Meaning	Observability
d(1)	*A damaged part is ready to be repaired*	*Observable*
d(2)	*A plate separated from the damaged part*	*Observable*
d(3)	*Grinding is finished in the right line*	*Observable*
d(4)	*Milling is finished in the right line*	*Observable*
d(5)	*Painting is finished in the right line*	*Observable*
d(6)	*Fitting is finished in the right line*	*Observable*
d(7)	*A slab separated from the damaged part*	*Observable*
d(8)	*Grinding is finished in the left line*	*Observable*
d(9)	*Milling is finished in the left line*	*Observable*
d(10)	*Painting is finished in the left line*	*Observable*
d(11)	*Fitting is finished in the left line*	*Observable*
d(12)	*One metallic plate inserted in the slab*	*Observable*
d(13)	*Buffer 1 is available*	*Observable*
d(14)	*Buffer 3 is available*	*Observable*
d(15)	*Painting is unavailable*	*Observable*
d(16)	*Pigment deficiency*	*Unobservable*
d(17)	*Buffer 2 is available*	*Observable*

## Data Availability

The data are contained within the article.

## Abbreviations

The following abbreviations are used in this manuscript:

DES	Discrete-event system
FSM	Finite-state machine
PN	Petri net
ILP	Integer linear programming
ILPP	Integer linear programming problem
